# Development of elastin-like polypeptide for targeted specific gene delivery in vivo

**DOI:** 10.1186/s12951-020-0574-z

**Published:** 2020-01-17

**Authors:** Aena Yi, Dahye Sim, Young-Jin Lee, Vijaya Sarangthem, Rang-Woon Park

**Affiliations:** 1 Department of Biochemistry and Cell Biology, Cell & Matrix Research Institute, Kyungpook National University, School of Medicine, Daegu, 41944 Republic of Korea; 2 Department of Pathology, All India Institute of Medical Sciences, New Delhi, 110029 India

**Keywords:** Gene delivery, AP1-ELPs, Tumor targeting, IL-4 receptor, ELP, Cell penetrating peptide

## Abstract

**Background:**

The successful deliveries of siRNA depend on their stabilities under physiological conditions because greater in vivo stability enhances cellular uptake and enables endosomal escape. Viral-based systems appears as most efficient approaches for gene delivery but often compromised in terms of biocompatibility, patient safety and high cost scale up process. Here we describe a novel platform of gene delivery by elastin-like polypeptide (ELP) based targeting biopolymers.

**Results:**

For better tumor targeting and membrane penetrating characteristics, we designed various chimeric ELP-based carriers containing a cell penetrating peptide (Tat), single or multiple copies of AP1 an IL-4 receptor targeting peptide along with coding sequence of ELP and referred as Tat-A_1_E_28_ or Tat-A_4_V_48_. These targeted polypeptides were further analyzed for its ability to deliver siRNA (Luciferase gene) in tumor cells in comparison with non-targeted controls (Tat-E_28_ or E_28_). The positively charged amino acids of these polypeptides enabled them to readily complex with negatively charged nucleic acids. The complexation of nucleic acid with respective polypeptides facilitated its transfection efficiency as well as stability. The targeted polypeptides (Tat-A_1_E_28_ or Tat-A_4_V_48_) selectively delivered siRNA into tumor cells in a receptor-specific fashion, achieved endosomal and lysosomal escape, and released gene into cytosol. The target specific delivery of siRNA by Tat-A_1_E_28_ or Tat-A_4_V_48_ was further validated in murine breast carcinoma 4T1 allograft mice model.

**Conclusion:**

The designed delivery systems efficiently delivered siRNA to the target site of action thereby inducing significant gene silencing activity. The study shows Tat and AP1 functionalized ELPs constitute a novel gene delivery system with potential therapeutic applications.

## Background

Gene therapy continues to show great potential as a means of treating various diseases including cancer [1–3]. For many decades RNAi (RNA interference) has provided a basic means of developing new classes of effective drugs that target the elimination of a specific pathogenic gene product or protein, and this strategy has been successfully applied to the treatment of cancer, infectious diseases, Parkinson’s disease, type-2 diabetes, and other diseases [4–10]. Many efforts have been made to ensure the success of this strategy, which centers on expressing genes of interest at specific locations and minimizing side effects. Successful gene delivery depends on the carrier used as the negative charges of naked siRNA, and oligonucleotides unable to pass through the cell membranes [11, 12]. Despite the clinical potential of RNAi, its degradation by RNA nucleases in vivo makes this approach inefficient under physiological conditions. Therefore, to achieve gene silencing, siRNA molecules must be delivered to specific cells and associated with RISCs (RNA-induced silencing complex) of the cytosol to degrades the target mRNA complementary to the single strand RNA so that it inhibits the pathogenic protein translation at the first place [13]. For these reasons, siRNA delivery systems that protect siRNA from degradation by serum proteins and enable the specific uptake or release of siRNA in cells or tissues with substantially reduce risks of systemic toxicity and immunogenicity are being activity investigated.

Primarily, virus-mediated gene delivery has been suggested to provide the most effective gene delivery systems, but is limited in terms of biocompatibility, patient safety, and high production costs [14]. Thus, non-viral approaches based on liposomes [15, 16], artificial cationic formulations [17, 18] and polymers [19, 20] have been used as alternatives, particularly to overcome the inherent immunogenicity of viral vectors [21, 22]. The polycationic polymer, PEI, is a commonly used non-viral vector because it efficiently binds to cell surfaces, and exhibits high uptake and endosomal escape [23]. But specialized polymer chemistry is required to synthesize functional cationic block copolymers and physicochemical properties (e.g., molecular weight and polydispersity) are difficult to control. Furthermore, specialized techniques are required to modify block polymers with targeting peptides or the confer different functional activities [24]. In fact, arginine-rich cell penetrating peptides (CPPs) have been shown to encapsulate siRNA efficiently due to electrostatic interactions, but these increase risks of collateral cytotoxicity [25–27].

Hence, successful gene expression requires highly biocompatible, target-specific delivery systems. The aptamer-integrated DNA nanostructure drug carriers was reported to target the tumor by the enhanced permeability and retention (EPR) effect and achieved improved cancer therapy [28]. In this study, a novel elastin-like polypeptide (ELP) based platform was devised for targeted non-viral gene delivery. The pharmacologic potentials of thermally sensitive ELPs were initially posited by Urry et al. and have been widely explored over the last decade [29]. They are composed of Val–Pro–Gly–Xaa–Gly (VPGXG) pentapeptide repeats, where ‘X’ is a guest residue that can be substituted by any amino acid except Pro [30, 31]. ELPs are water-soluble, biocompatible, non-toxic biopolymers [19] that exhibit reversible temperature phase transition, which is dependent on the nature of the guest residue, ionic state, and molecular weight [32]. ELPs are highly soluble in aqueous solutions below their transitions temperatures (Tt) but insoluble above. They are synthesized using genetic-engineering methods, which mean their physical and chemical properties can be precisely controlled [33]. Thus, varying amino acid composition by including more positively charged residues in ELPs increases stability under physiological conditions. In fact, ELPs fused to a cell penetrating peptide (CPP) have been utilized as promising vehicles for delivering drugs and therapeutic peptides into solid tumors [34, 35].

Here, we have constructed an ELP-based tumor targeting carrier Tat-A_1_E_28_ or Tat-A_4_V_48_ containing two complementary features, that is, efficient cell penetration and cell type specificity, by incorporating Tat (a CPP) and single or multiple copies of AP1 (an IL-4 receptor specific ligand). Despite being considered good candidate siRNA carriers, Tat peptides lack cell-type specificity, and hence AP1 was incorporated. AP1 is an atherosclerotic plaque and breast tumor tissue homing peptide that selectively binds to interleukin-4 receptors (IL-4Rs), which were discovered using a phage screening method [36]. Many researches have confirmed that IL-4Rs are highly expressed in a wide variety of human tumors, including renal cell carcinoma, squamous cell carcinoma of the head and neck malignant glioma, AIDS-associated Kaposi’s sarcoma, and several breast cancer cell lines [37–40]. Therefore, in this study the mechanism of gene delivery (siRNA for luciferase gene) by ELP-based tumor targeting carriers were studied in terms of cellular uptake and payload release and expression in vitro and in vivo. The pattern of accumulation in tumor, as well as bio-distribution between single verses multivalent based ELP was investigated in vivo.

## Materials and methods

### Cell culture

MDA-MB231 cells (a human breast cancer cell line) and 4T1-luc cells (a murine breast cancer cell line) were obtained from American Type Culture Collection (ATCC). MDA-MB231 cells were grown in Dulbecco’s modified Eagle’s medium (DMEM) (Hyclone, Invitrogen, Carlsbad, CA, USA), and 4T1-luc cells in RPMI (Roswell Park Memorial Institute medium) (Hyclone, Invitrogen Carlsbad, CA, USA) containing 10% fetal bovine serum (Gibco, Canada) and 100 U/mL penicillin and 100 µg/mL of streptomycin (Sigma Aldrich). Cells were maintained at 37 °C in a humidified 5% CO_2_ atmosphere.

### Thermal characterization

All the polypeptides (E_28_, Tat-E_28_, Tat-A_1_E_28_ and Tat-A_4_V_48_) were diluted to 25 µM with phosphate saline-buffered (PBS) and their transition temperatures (Tt) were determined by monitoring turbidity profiles at 350 nm between 20 and 55 °C using UV–visible spectrophotometer. The absorbance was monitored while increasing temperature at 1 °C/min.

The phase transition of respective polypeptides after complexation with siRNA was further analyzed. To determine, 10 µM of siRNA was mixed with 200 µM ELP variants (molar ratio 1:20; siRNA: protein) and incubated for 20 min at room temperature to form siRNA/ELP complexes. Tt values were determined at 350 nm from 20 to 55 °C using a UV–visible spectrophotometer.

### Gel retardation assay

In order to assess the ability of nucleic acid encapsulating ability of respective polypeptides, a gel retardation assay was performed. 10 µM of siRNA was mixed with different concentrations of ELPs at molar ratios ranging from 1:5 to 1:30 (siRNA: ELPs) at a constant final reaction mixture volume (40 μL) and incubated for 20 min at 25 °C. The siRNA/ELP variant complex formation was confirmed by 1% agarose gel electrophoresis [41, 42].

### Determination of particle sizes and surface charges

siRNA/ELP complexes were prepared at a molar ratio of 1:20 (siRNA:ELPs), as described above. Complex particle size distributions and zeta potentials were determined using an ELS-Z2 (Otsuka Electronics Korea Co, Ltd., Korea). Shapes and sizes of nanocomplexes were determined by transmission electron microscopy (TEM; CM30 Electron Microscope, Philips, CA). siRNA/ELP complexes were dropped on the TEM grid, allowed to dry for 10 min, negatively stained with 2 wt% uranyl acetate solution, and observed in the TEM.

### RNase A stability test

ELP variants were mixed with 10 µM of NC-siRNA (negative control) at molar ratio of 1:20 (siRNA:ELPs) in a final volume of 40 μL with or without 0.625 µg of RNase A and incubated for 6 h at 37 °C. Heparin sodium (1 µL; JW Pharmaceutical, S. Korea) was then added for 5 min to disassemble complexes and subjected to 1% agarose gel electrophoresis in TBE buffer [14, 42].

### Flow cytometry analysis

To access cell binding activities, Alexa-488 C5 maleimide labeled ELPs (0.3125 μM) were incubated with MDA- MB231 or 4T1 cancer cell, which are known to express IL-4 receptor, for 1 h at 4 °C. Cells were then washed with PBS and cell binding was analyzed by flow cytometry (Becton–Dickinson, San Jose, CA). For the analysis, 10,000 events were collected per sample.

### Intracellular tracking

MDA-MB231 cells (8 × 10^4^) were seeded in 4-chamber slide and incubated with anti-Early endosome antigen 1 (BD Biosciences, Franklyn Lakes, NJ, USA) diluted in DMEM (Dulbecco’s Modified Eagle Medium) for 1 h at 37 °C. Cells were then washed with PBS several times to remove unbound antibody and then incubated with Alexa Flour-594 goat anti-mouse IgG (Life Technologies Corporation, Eugene, OR, USA) secondary antibody and siRNA/ELP variant complexes (50 pmol NC-siRNA labeled with Alexa 488) for 1 h. Cells were then fixed with 4% paraformaldehyde and nuclei were stained with Hoechst 33342. The internalizations of siRNA/ELP complexes into early endosomes were assessed by confocal microscopy (LSM700; Carl Zeiss).

### Luciferase assay

4T1-luc cells (stably express luciferase) were seeded on 96 well plates and grown for 24 h. Different concentrations (50, 100, or 200 nM) of negative control siRNA (NC siRNA; MW-13284.8) and luciferase siRNA (siRNA-1; MW-13398.9) were encapsulated in ELPs at a molar ratio of 1:20. Cells were then treated with siRNA/ELP complexes for 6 h and media were replaced with complete media (RPMI containing 10% FBS). After 24 h of incubation, 3 µL of luciferin substrate was added to each well and luciferase activity was measured using the in vivo imaging system, IVIS (Perkin Elmer). Maximum siRNA deliveries by ELPs were determined by comparing luciferase activity versus negative control siRNA.

### In vivo fluorescence imaging

All animal experiments were conducted according to the guidelines issued by the Animal Care and Use Committee of Kyungpook National University (Permit Number KNU 2019-34). Wild type BALB/c mice (females/5 weeks old) were housed in a specific pathogen-free environment. Tumors were created by subcutaneously injecting 4T1 cells (5 × 10^6^ cells) into right flanks. Tumors of appropriate size (200–400 mm^3^) usually developed within 1 week. Mice bearing a subcutaneous tumor were anesthetized with 1.5% isoflurane inhalation and injected with Cy5.5 labelled siRNA encapsulated in ELP variants intravenously at a molar ratio of 1:20 (siRNA:ELP variant) at a dose of ~ 250 µg/kg. In vivo fluorescence images were taken at different times using in vivo imaging system IVIS. For ex vivo analysis, animals were euthanized with CO_2_ and tumors and organs were collected six hours post-injection. Ex vivo fluorescence images were obtained using IVIS. For immunohistological analysis, tumor tissues were fixed with 4% paraformaldehyde overnight and frozen for cryosectioning. Tissues were sectioned at 8 mm, incubated with IL-4R antibody (R&D Systems; 1:100) overnight at 4 °C and observed under a confocal microscope after stained with Alexa 488 labeled goat anti mouse IgG secondary antibody (1:200) and DAPI.

### In vivo gene silencing

Female wild type BALB/c mice were implanted with 4T1-luc cells (stably expressed the luciferase gene) as described above. 5 days after implantation of 4T1-luc, the mice were divided into five groups (n = 10 per group). Treatment was initiated by daily intravenous injection of respective siRNA/ELP variant complexes (250 µg/kg siRNA) or PBS (control) for 6 days. Luciferase gene silencing due to ELPs-mediated siRNA delivery was monitored by observing luciferase bioluminescence using IVIS every other day for 8 days. Luciferase gene silencing was also confirmed histologically. On day 8, tumor tissues were extracted and rapidly frozen for cryosectioning. Tumor Sects. (8 mm thick) were stained with anti-Luc antibody (1:100) and counterstained with Alexa 488 labeled goat anti-mouse IgG secondary antibody (1:200) and observed under a confocal microscope.

### Statistical analysis

The significances of differences between experimental and control groups were determined using the Student’s *t* test for two groups or by one-way analysis of variance (ANOVA) for more groups. Statistical significance was accepted for p values of P < 0.05, and is denoted by asterisks in figures.

## Results

### Design of ELPs and physical characterization

**Fig. 1:**
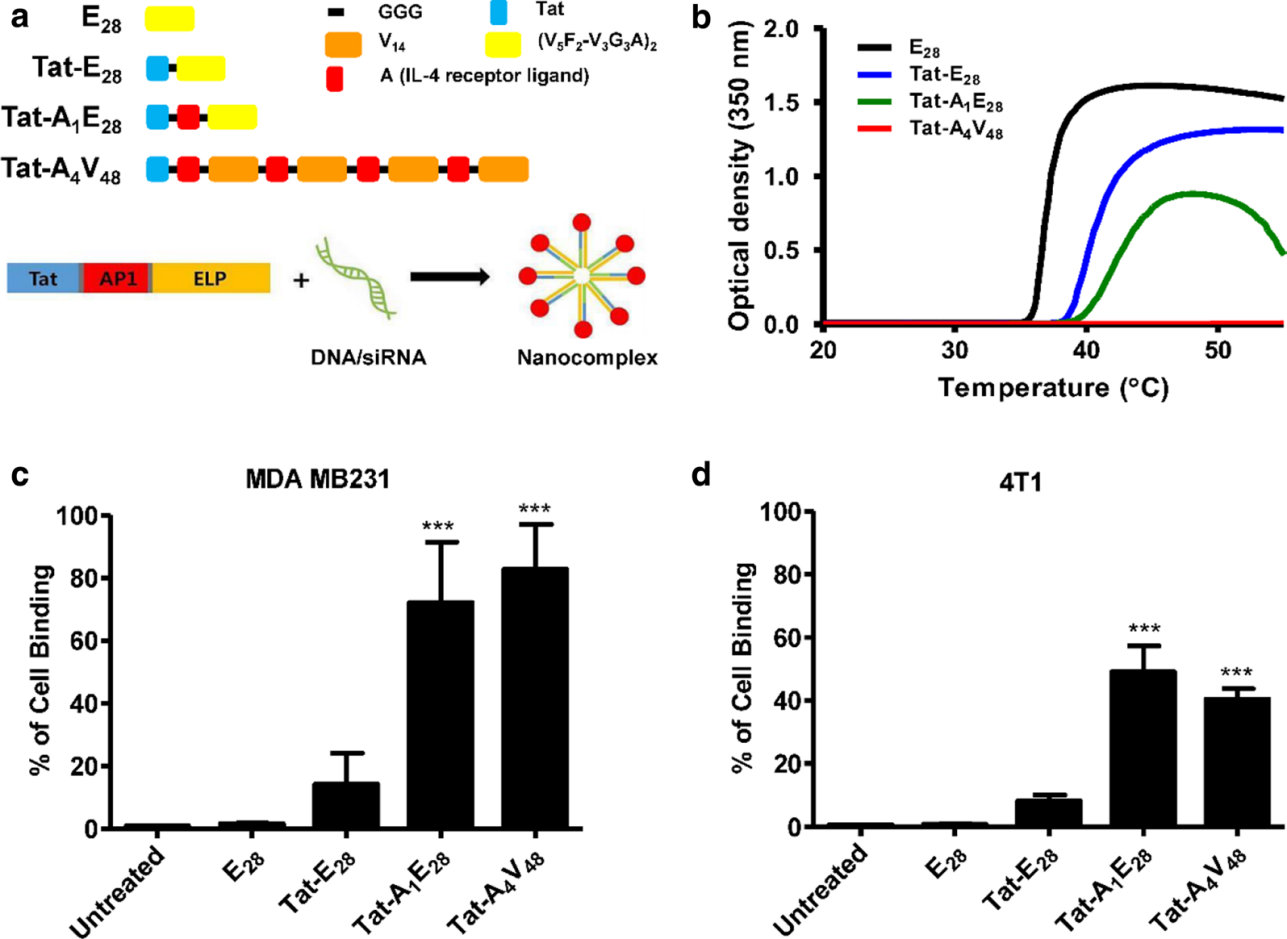
Characterization of ELP variants. **a** Schematic representation of the amino acid sequences of the designed variants (E_28_, Tat-E_28_, Tat-A_1_E_28_, and Tat-A_4_V_48_). The positively charge amino acids present in ELPs induced complexation with negatively charged nucleic acids to form nanoparticle like structures. **b** Turbidity profiles of ELPs determined by measuring O.D. values at 350 nm while increasing temperature at 1 °C/min. Cell binding activities of the ELPs (0.3125 µM) were measured by flow cytometry after incubating MDA MB231 (**c**) or 4T1 (**d**) cells with the respective polypeptides labeled with Alexa-488 at 4 °C. Results are presented as means ± SDs (n = 6). ***P < 0.0001 (Student’s t-test), significant difference for Tat-A_1_E_28_, and Tat-A_4_V_48_ compared with E_28_

For targeted gene delivery, tumor specific highly intracellular penetrable ELPs were constructed by combining coding sequence of Tat (cell penetrating peptide, CPP) and IL-4 receptor specific targeting ligand (AP1) along with ELP sequence (Fig. 1a and Additional file 1: Figure S1). Two ELP variants, that is, Tat-A_1_E_28_ (A_1_ represent one-unit AP1and E_28_ represent repeat of [(VPGVG)_5_(VPGFG)_2_-(VPGVG)_3_(VPGGG)_3_ (VPGAG)]_2_) and Tat-A_4_V_48_ (V_48_ indicates 48 pentapeptide repeat of VPGVG) were used as tumor specific delivery system. Tat-E_28_ and E_28_ were used as non-targeted controls. It was believed, the positive amino acid (arginine, R) in ELP variants (ELPs) would facilitate nucleic acid condensation, cause nano-complex formation (Fig. 1a), and thereby shield genetic material from degradation. ELP sequences with pentapeptide repeat (Val–Pro–Gly–Xaa–Gly), in which the Xaa residue was phenylalanine (F) was used to obtain ELP of lower molecular weight with transition temperatures (Tt) near body temperature. AP1 was positioned on the C-terminus of Tat and was followed by the sequence of ELP. For Tat-A_4_V_48_, four copies of AP1 peptide were first incorporated with ELP using the recursive directional ligation (RDL) method [33], and later linked to the C-terminus of Tat. A three-component glycine spacer (GGG) was incorporated between Tat and AP1 to minimize the risk of interference. The designed polypeptides were expressed in Escherichia coli and purified by ITC (inverse temperature cycling), which resulted in high purities after 4 cycles. Protein purities and molecular weights were confirmed by SDS-PAGE and copper chloride (0.3 M) staining (Additional file 2: Figure S2). SDS-PAGE revealed molecular weights were approximate with theoretical MWs based on amino acid contents as follows; E_28_ (12.18 kDa), Tat-E_28_ (13.19 kDa), Tat-A_1_E_28_ (15.00 kDa), and Tat-A_4_V_48_ (26.81 kDa). Interestingly, additional bands with molecular weights twice those expected for the ELP variants were observed indicating dimer formation due to presence of cysteine residue at C-terminal.

The thermal behaviors of the ELPs were monitored by measuring turbidity profiles at 350 nm (OD_350_) while increasing temperature at 1 °C/min (Fig. 1b). T_t_ values (defined as the temperature at which turbidity of a protein solution reached 50%) were determined at a concentration of 25 μM in PBS. The Tt values of E_28_, Tat-E_28_, and Tat-A_1_E_28_ were ~ 37 °C, 40.5 °C, and 42.3 °C, respectively. Coacervate formation was not observed for Tat-A_4_V_48_, even at a concentration of 50 μM, indicating that it’s greater hydrophilicity shift its phase transition to a higher temperature. The T_t_ values of the other ELPs were just above body temperature. Temperature-dependent increases in ELPs particle sizes were analyzed by DLS (dynamic light scattering). The particle sizes of E_28_, Tat-E_28_, Tat-A_1_E_28_, and Tat-A_4_V_48_ were 390, 78.1, 30.1 and 29 nm, respectively (Additional file 11: Table S1).

### Analysis of cell-binding activities

To investigate the binding activity of ELPs in vitro, MDA MB231 human breast cancer or 4T1 murine cells were incubated with Alexa-488 labeled ELPs (0.3125 µM) for 1 h at 4 °C. Cell binding activities, as determined by flow cytometry, revealed that Tat-A_1_E_28_ and Tat-A_4_V_48_ had higher cell binding activities than non-targeting controls (E_28_, Tat-E_28_). The cell binding percentages of Tat-A_1_E_28_ and Tat-A_4_V_48_ were 72.09% and 82.74% in MDA MB231cells (Fig. 1c). Whereas Tat-E_28_ and E_28_ displayed minimal cellular accumulations of 14.1% and 1.6%, respectively. In 4T1 cells, the binding percentages of Tat-A_1_E_28_ and Tat-A_4_V_48_ were 49.04% or 40.89% (Fig. 1d), and E_28_ and Tat-E_28_ displayed lower binding activities of 0.89% and 8.09%. In-line with flow cytometry findings, confocal microscopy showed Tat-A_1_E_28_ and Tat-A_4_V_48_ had higher cell binding abilities to both MDA MB231 (Additional files 3 and 4: Figure S3 and S4) and 4T1 (Additional files 5 and 6: Figure S5 and S6) cells. Interestingly, Tat-A_4_V_48_ achieved higher cellular accumulations and uptakes than other ELPs.

### Encapsulation of siRNA with ELP variants and physical characterization

**Fig. 2:**
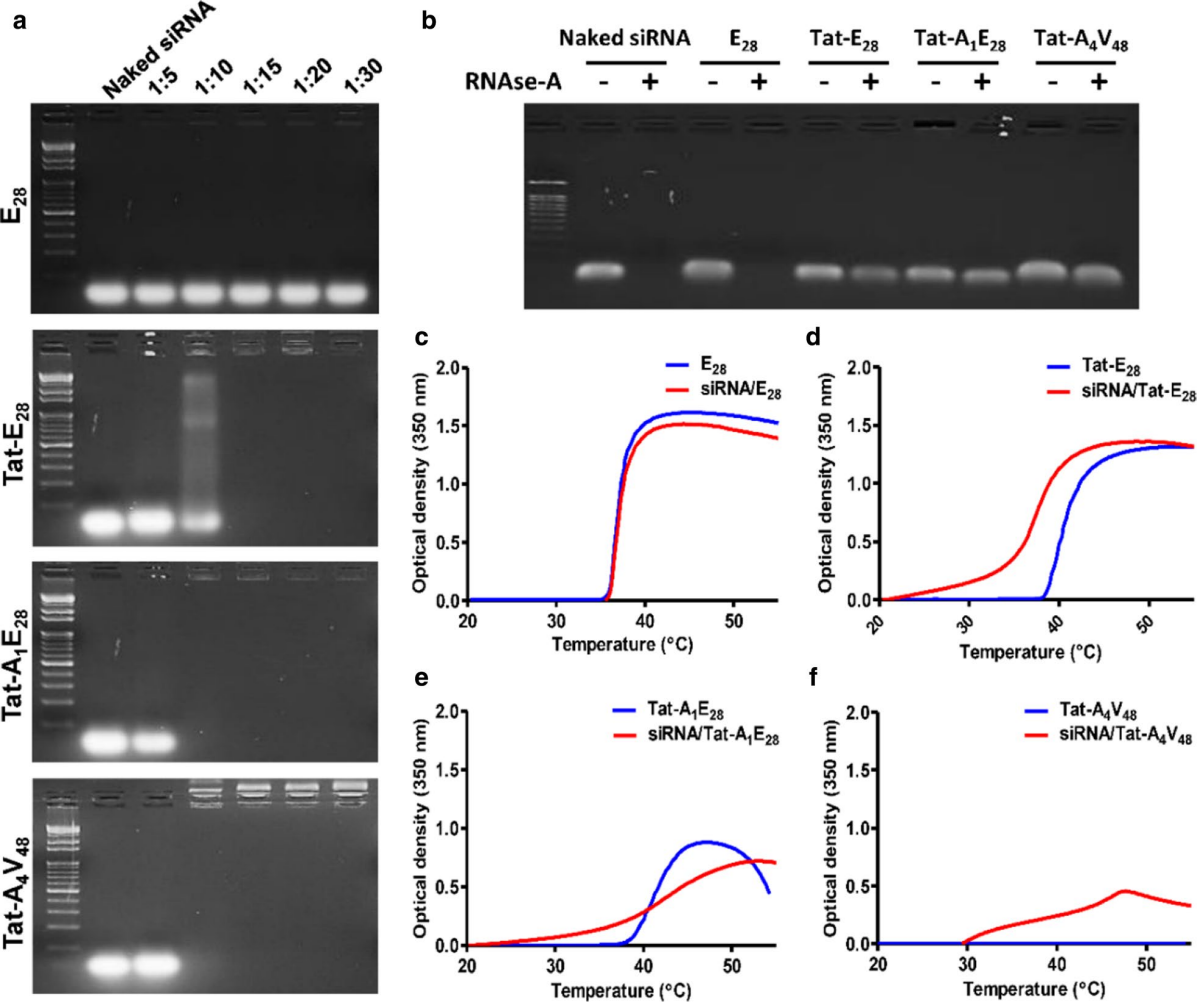
Gel retardation assay of siRNA/ELPs complexes. **a** Condensations of siRNA (10 µM) and ELPs (50–300 µM) were monitored at molar ratios from 1:5 to 1:30 (siRNA: ELPs). **b** Stability of encapsulated siRNA was determined by treating siRNA/ELP complex with or without RNase A for 6 h. **c**–**f** Changes in thermal behaviors of resulting siRNA/ELP complexes were compared with naïve ELPs by measuring turbidity profiles at 350 nm while increasing temperature at 1 °C/min

**Fig. 3:**
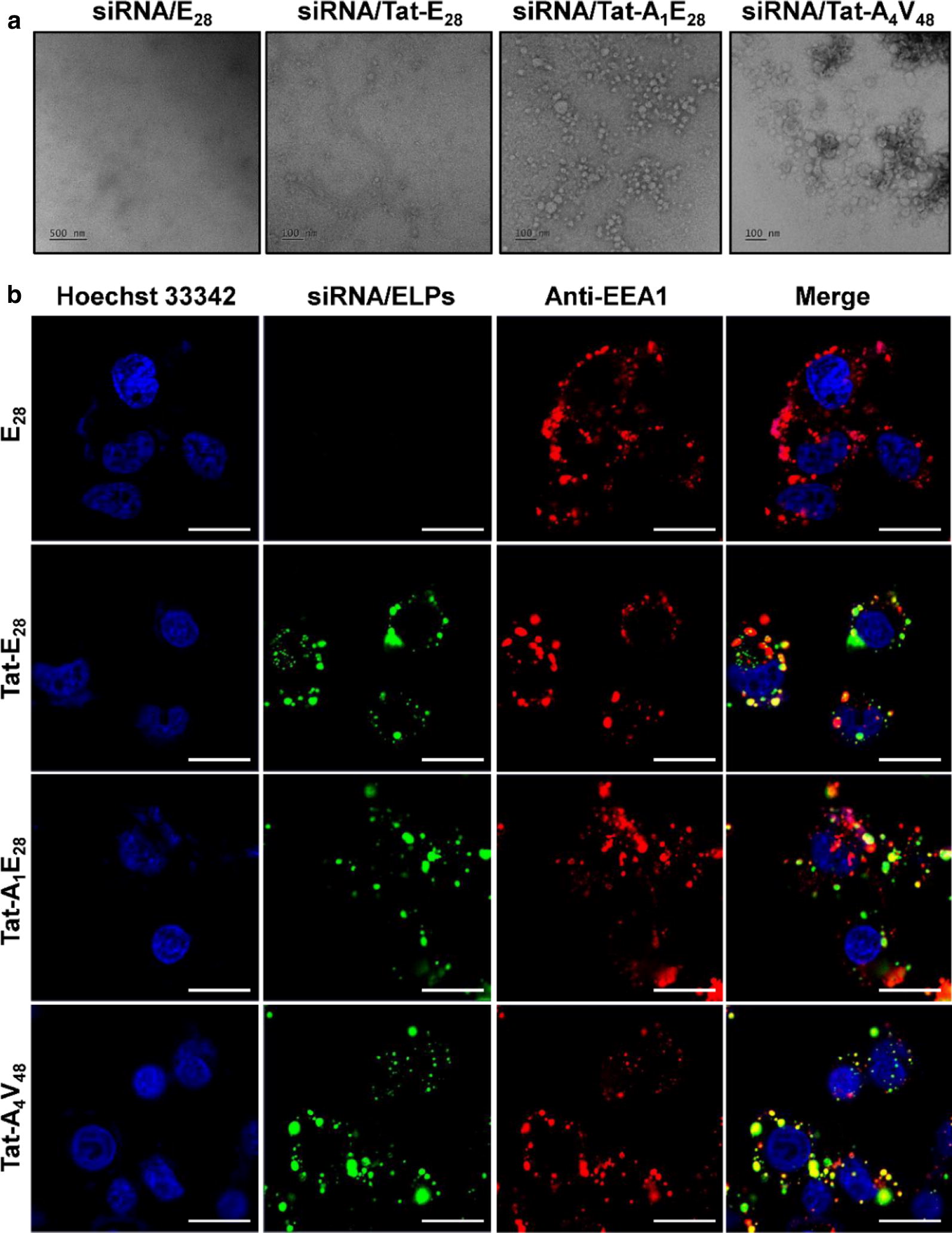
Sizes and structural analysis of siRNA/ELPs complexes and intracellular trafficking. **a** TEM images of nanoparticle-like structures formed by siRNA/ELPs complexes at room temperature. Scale bar = 100 nm. **b** In order to determine subcellular localizations and siRNA release, MDA MB231 cells were stained with anti-EEA1 (early endosome antigen) after incubating them with siRNA/ELP complexes for 1 h at 37 °C. The cellular localizations of complexes in early endosomes were observed by confocal microscopy. Representative confocal microscopic images of five experiments. Blue, nuclei stained with Hoechst; Green, siRNA/ELP variant complexes; Red, Anti-EEA1. Scale bar, 10 µm

Similarly, ELPs readily condensed siRNA at molar ratio ranges from 1:5 to 1:30 (siRNA: ELP variant). Maximum encapsulation of siRNA was observed at a molar ratio of 1:10 for Tat-A_1_E_28_ and Tat-A_4_V_48_ (Fig. 2a), indicating the presence of positively charge amino acids in Tat and AP1 bound to the negatively charged siRNA backbone promoted encapsulation. Whereas Tat-E_28_ showed complete siRNA encapsulation at a molar ratio of 1:15, and E_28_ did not encapsulate siRNA. To examine siRNA/ELP complexes stabilities, complexes were treated with RNaseA for 6 h and siRNA degradation was examined after heparin sodium disassembly. Agarose gel electrophoresis shown that siRNA in siRNA/E_28_ was degraded as easily as naked siRNA (Fig. 2b). The minimum degradation of the siRNA was observed for Tat-E_28_ and Tat-A_1_E_28_ while no changes were seen in Tat-A_4_V_48_ encapsulated siRNA. Thus, Tat-A_4_V_48_ was found to encapsulate siRNA properly and protect effectively from RNase attack. Simultaneously, observation of turbidity profile of siRNA/ELP complexes displayed slightly reduced Tt compared to naked ELPs (Fig. 2c–f). The average particle diameters of siRNA complexes with Tat-E_28_, Tat-A_1_E_28_ and Tat-A_4_V_48_ were 868.3, 534, and 247.4 nm respectively. There were minimum changes in the surface charge of the ELPs after complexation with siRNA. The sizes and shapes of the complexes formed were examined by transmission electron microscopy (TEM). The images obtained showed that Tat-A_1_E_28_ and Tat-A_4_V_48_ complexes appeared as disperse or aggregates of spherical nanostructures with overall diameters ranging from 10 to 85.7 nm (Fig. 3a) whereas Tat-E_28_ were exist as disordered structures. No particle formation was observed for E_28_ complexes.

### Intracellular localization of siRNA/ELPs complexes

The cellular entry and sub-cellular localizations of siRNA/ELP variants was assessed in MDA MB231 cells by confocal microscopy. Alexa-488 labeled siRNA was used to visualize internalization patterns directly. Confocal images taken 1 h after siRNA/ELP complexes treatment revealed the co-localization of complexes (green) with endosomes or lysosomes (Red). Tat-A_1_E_28_ and Tat-A_4_V_48_ co-localized most with early endosomes (EEA1) (Fig. 3b) and lysosomes (Additional file 7: Figure S7) as evidenced by yellow spots resulting from overlapping green and red signals of EEA1 and lysosomes. This clearly indicated that siRNA/ELP complexes were endocytosed in the cell, localized in endosome followed by lysosomes. Later it might disrupt the endosomal or lysosomal membrane which in turn released the siRNA at target site either in cytoplasm or translocate into nucleus. On the other hand, Tat-E_28_ showed minimal localization, and no uptake or co-localization was observed for E_28_.

### Determination of gene silencing by luciferase assay

**Fig. 4:**
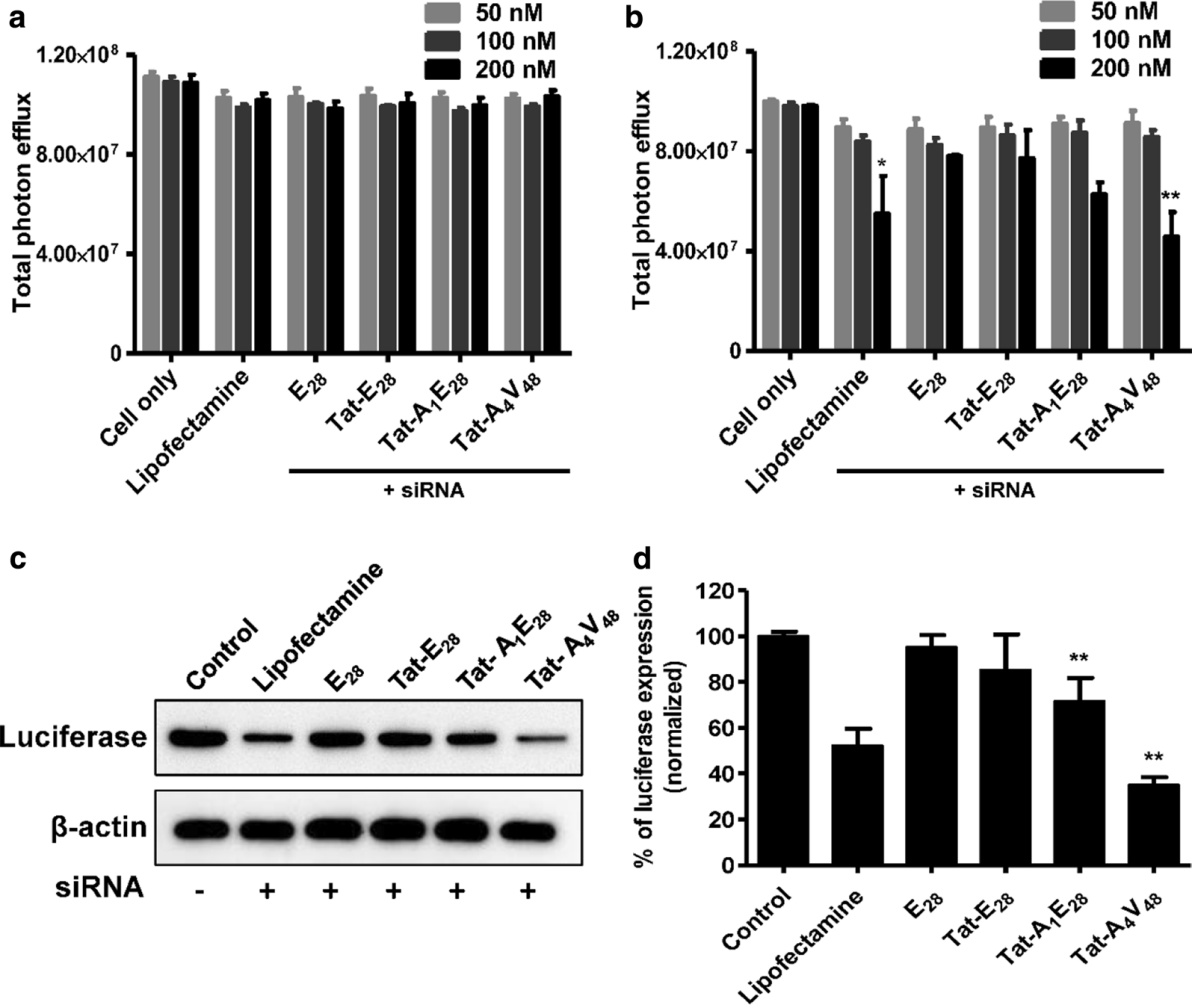
In vitro gene silencing. **a** Negative control siRNA (NC-siRNA) or **b** siRNA for luciferase genes (at 50, 100, or 200 nM) were mixed with respective ELPs. 4T1-luc cells were transfected with siRNA/ELPs complexes for 3 h and checked for luciferase gene silencing after 48 h of transfection using an IVIS. Results are presented as the means ± SDs of three separate experiments performed in triplicate. A t-test was performed to determine the significance of various groups after siRNA/ELPs complex treatments with different concentration of siRNA (50, 100, 200 nM). *P < 0.05, Control versus lipofectamine mediated siRNA (200 nM) delivery and **P < 0.001, Control versus siRNA/Tat-A_4_V_84_ mediated siRNA (200 nM) delivery. **c** 4T1 cells were transfected with siRNA/ELP variant complexes for 3 h and harvested at 48 h after transfection. Later cell lysates were western blotted to assess luciferase gene silencing. **d** The histogram of normalized luciferase band intensities obtained using image J software. **P < 0.001 (Student’s t-test), significant difference for Tat-A_1_E_28_ or Tat-A_4_V_84_ compared with control

The abilities of E_28_, Tat-E_28_, Tat-A_1_E_28_ and Tat-A_4_V_48_ to deliver genes were investigated by examining target gene silencing efficacy in vitro. Various concentration of siRNA (50, 100, or 200 nM) were encapsulated using ELPs and transfected into 4T1 cells stably expressing luciferase (Luc). All the ELPs such as Tat-E_28_, Tat-A_1_E_28_ and Tat-A_4_V_48_ showed the knock down of luciferase gene in dose dependent manner (Fig. 4b and Additional file 8: Figure S8b). Comparatively Tat-A_4_V_48_ displayed significant silencing of luciferase expression by 53% in 200 nM siRNA concentration. No change in luciferase gene expression was observed when cells were transfected with siRNA/E_28_ or NC-siRNA/ELPs complexes (Fig. 4a and Additional file 8: Figure S8a). Later western blot analysis of 4T1-luc cells collected after 48 h transfection with siRNA/Tat-A_4_V_48_ revealed a knockdown efficiency of 65% at a molar ratio of 1:20 (siRNA concentration 200 nM) (Fig. 4c and d). However, siRNA**/**Tat-A_1_E_28_ or Tat-E_28_ complexes suppressed luciferase gene expression by 28.4 or 14.8%, respectively. On the other hand, Lipofectamine 2000/siRNA (positive control) only knocked down targeted gene expression by 48%. No change in luciferase gene expression was observed in siRNA/E_28_ transfected cells. As the main purposes of ELPs were successfully delivered the gene with minimal cytotoxicity, hence toxicity of complexes was assessed through cell viability assay. The viability of the cells was reduced by 30% at 200 nM siRNA concentration by lipofectamine 2000 treatment, whereas cell viability was unaffected after treatment with siRNA/ELP complexes (Additional file 8: Figure S8c).

### In vivo tumor targeting activity and gene silencing by siRNA/ELPs complexes

**Fig. 5:**
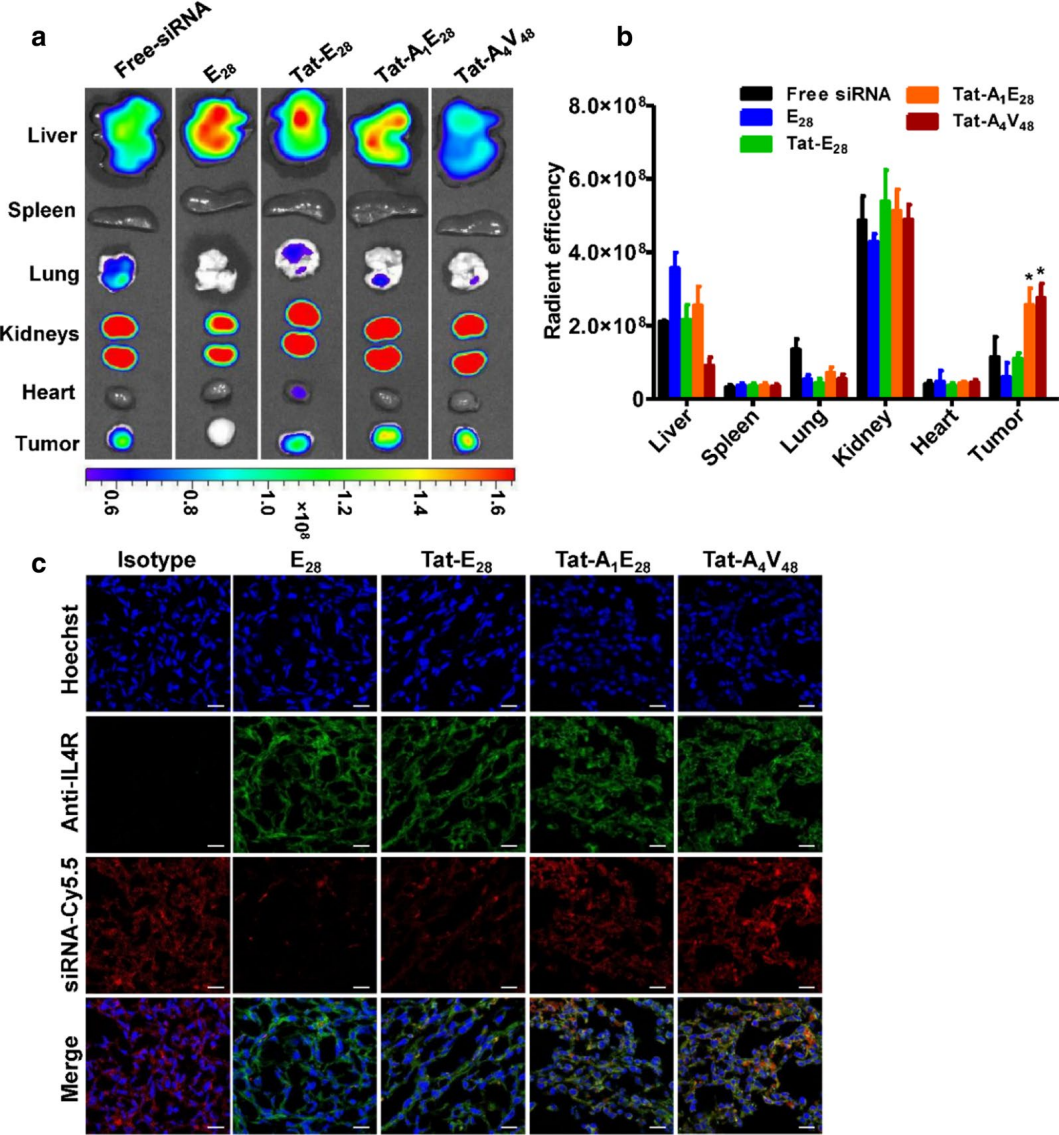
In vivo biodistribution imaging of siRNA/ELP variant complexes. **a** For in vivo IVIS imaging, Cy5.5 labelled siRNA (5 µg) was encapsulated by 200 µM of the ELPs. SiRNA/ELPs complexes were intravenously injected to 4T1 tumor bearing Balb/c wild type mice (n = 10). The tumors and organs were excised at 6 h post administration and fluorescence intensities were determined by IVIS imaging. Representative optical images of three independent experiments. **b** Quantification of the ex vivo fluorescence intensities of tumors and other organs at 6 h after injection (n = 10). *P < 0.05 (Student’s t-test), significant difference for Tat-A_1_E_28_ or Tat-A_4_V_48_ compared with free-siRNA. **c** Histological analysis of the localizations of siRNA/ELP variant complexes (red) in tumors at 6 h after injection. Nuclei were stained with Hoechst (blue), and IL-4R expression on cells was visualized by anti-IL-4 receptor antibody staining (green). The confocal images are representative of three experiments (Scale bar, 20 μm)

For targeted gene therapy, all carriers should shield genetic material from biological protagonists. Accordingly, we investigated the tumor specific deliveries of Cy5.5 labelled siRNA by the different ELPs in a 4T1 allograft model, generated by injecting tumor cells subcutaneously into the right flanks of female BALB/C wild type mice. IVIS images taken at different times after intravenously injecting of siRNA/ELPs complexes showed that Tat-A_1_E_28_ and Tat-A_4_V_48_ rapidly distributed and time-dependently accumulated in tumors. In contrast Tat-E_28_ and E_28_ showed lower accumulations in tumor tissues and high level in others organs (Additional file 9: Figure S9). Isolated organ imaging at 6 h post-administration indicated that siRNA/Tat-A_1_E_28_ and siRNA/Tat-A_4_V_48_ at a molar ratio of 1:20 exhibited higher fluorescence intensities in tumors than non-targeting ELPs (Fig. 5a and b). However, fluorescence intensities were strong in kidneys in all cases indicating rapid metabolism and excretion from the body through the renal route. Histological examination of tumor cryosections confirmed significant accumulations of Tat-A_1_E_28_ and Tat-A_4_V_48_ in tumor tissues. The robust red signals, representing Cy5.5-labeled siRNAs, were localized between blue and green areas, which represented nuclei and tumor cells, respectively (Fig. 5c). These observations confirmed that siRNAs were delivered into the cytoplasm of the tumor cells, where most likely RNA interference happens.

**Fig. 6:**
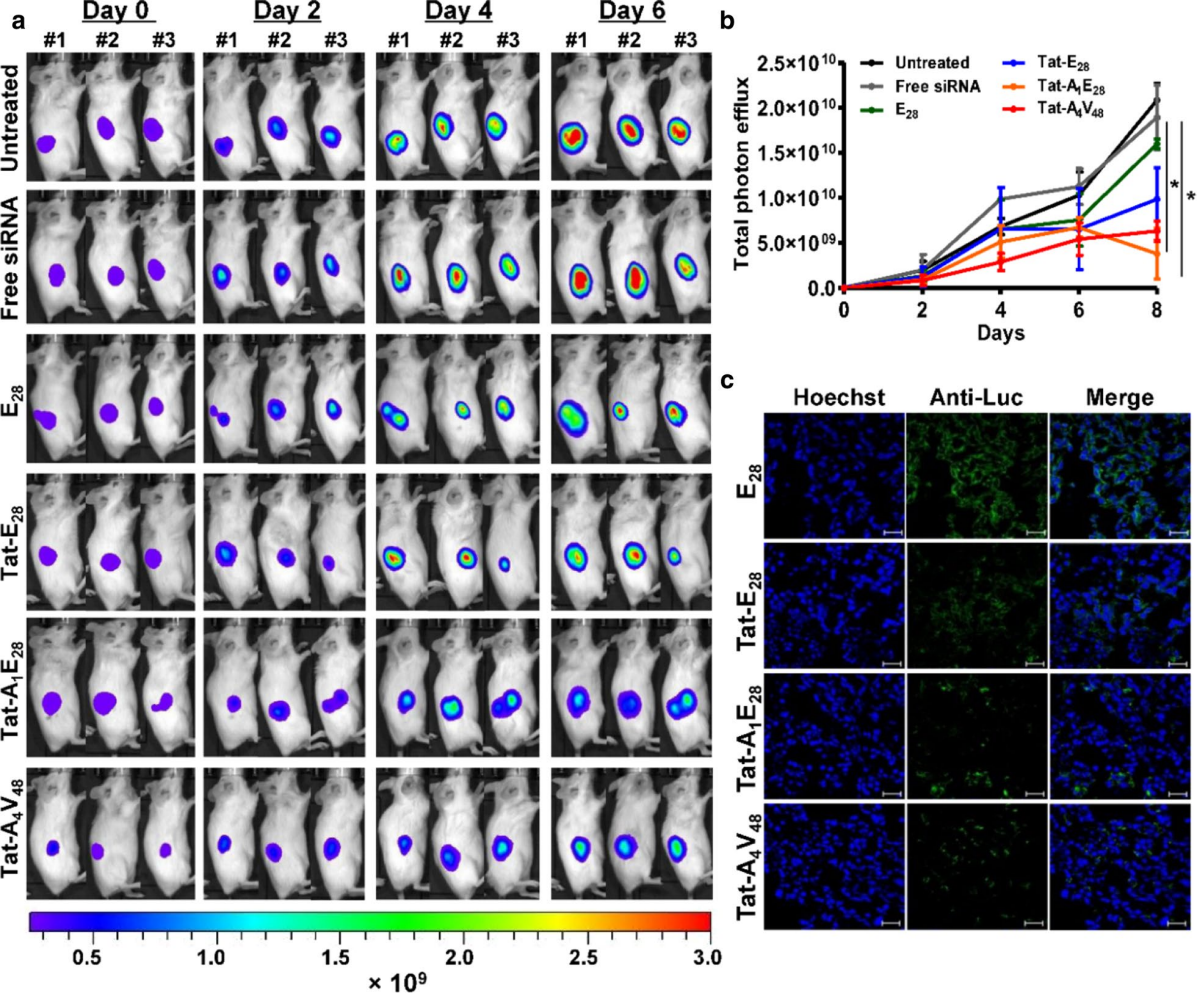
In vivo gene silencing by siRNA/ELP variant complexes. **a** For in vivo gene silencing, siRNA-1 (5 µg) was encapsulated using 200 µM of ELP variants and administered by intravenous injection to Balb/c wild mice (n = 10) bearing a 4T1 tumor daily for 6 days. Time-dependent reductions in bioluminescence intensities of subcutaneous tumors were determined by IVIS imaging. **b** Quantification of tumor bioluminescence intensities at different times (n = 10). *P < 0.05 (Student’s t-test), significant difference for Tat-A_1_E_28_ or Tat-A_4_V_48_ compared with free-siRNA. **c** After the 6-days treatment period tumor tissues were excised and tissue sections were stained with anti-Luc antibody. After nuclear staining with DAPI, tissue sections were observed under a confocal microscope. Hoechst, blue; and anti-Luc antibody, green. The confocal images are representative of three experiments (Scale bar, 20 μm)

Further it was observed that daily intravenous injection of siRNA/Tat-A_1_E_28_ or Tat-A_4_V_48_ suppressed the expression of luciferase significantly as compared with siRNA/Tat-E_28_ (Fig. 6a and b). On the other hand, tumors in the PBS and siRNA/E_28_ treated groups displayed vigorous increases in bioluminescence intensity. Immunohistological examination of tumor tissue further confirmed that Tat-A_1_E_28_ and Tat-A_4_V_48_ facilitated siRNA accumulation and uptake by tumor cells and significantly silenced the Luciferase gene in vivo (Fig. 6c). Whereas minimal or no silencing was observed in the tumor tissue obtained from siRNA/E_28_, free siRNA, or PBS treated mice (Fig. 6c and Additional file 10: Figure S10).

## Discussion

The stabilities of siRNA in vivo are major concerns of those charged with the development of targeted specific gene delivery systems as they markedly influence cellular uptake and endosomal escape. ELPs due to various favorable properties, it is considered as a promising candidate of gene delivery. For example, the cross-linking of ELP with lysine protects siRNA from being degraded by intracellular nucleases in cytoplasm. Free ELP does not exhibit any intrinsic cytotoxicity, which is a major consideration when designing siRNA carriers [43]. Furthermore, unlike many synthetic carriers, ELPs are composed of amino acids that can be degraded into non-toxic metabolites in vivo [44]. Finally, ELP can be easily designed and synthesized by cloning and easily modified, for example, changes in its MW and chemical properties can be used to prolong the plasma half-life of siRNA.

Here, we describe the development of an ELP-based gene carrier comprised of a highly hydrophobic ELP (VPGFG) residue conjugated with Tat and AP1 at its N-terminal (Fig. 1a). Various ELPs (E_28_, Tat-E_28_, Tat-A_1_E_28_ and Tat-A_4_V_48_) were designed to complex with siRNA due to electrostatic interaction with the poly-arginine (R) residue present in Tat or AP1. The AP1 ligand was use to target IL-4R expressing tumor cells, and ELP was chosen due to its stability and thermal sensitivity. Cell penetrating peptide, Tat was use to have highly membrane translocation properties and nuclear localization functions. Confirmation of proteins sizes by SDS-PAGE revealed they were successfully expressed at high yields. Thermal characterization of ELPs revealed that Tat-E_28_ and Tat-A_1_E_28_ had Tt values of 40.5 °C and 42.2 °C (Fig. 1b), respectively, that is, just above body temperature, which is considered suitable for clinical applications. On the other hand, Tat-A_4_V_48_ had a higher Tt and exhibited minimal transition and high solubility, which were attributed to the multiple copies of hydrophilic AP1 it contained.

The presence of AP1 (the targeting ligand) on the ELP backbone improved cellular binding, which is required for tumor targeting ability. In vitro flow cytometry demonstrated Tat-A_1_E_28_ and Tat-A_4_V_48_ bound to MDA MB231 human breast and 4T1 murine cancer cells markedly more than E_28_ and Tat-E_28_, which exhibited minimal binding (Fig. 1c and d). So, this result verified that cellular binding depended more on the presence of IL-4R on the surfaces of tumor cells than on nonspecific penetration by Tat or the effect of hyperthermia. In-line with these observations, confocal imaging showed obvious cellular accumulations and uptakes of Tat-A_1_E_28_ and Tat-A_4_V_48_ but minimal accumulations and uptakes of E_28_ and Tat-E_28_ in IL-4R expressing tumor cells, which indicated that these enhancements were due to the presence of AP1.

Our investigations on the condensations of siRNA with Tat-E_28_, Tat-A_1_E_28_ and Tat-A_4_V_48_ revealed successful encapsulation at different molar ratios (Fig. 2a). Positive charged amino acids in Tat bind with negatively charged siRNA. Condensation at a lower molar ratio, as displayed by Tat- A_4_V_48_, may have been due to a greater positive charge of the arginine residue in AP1. Encapsulation of siRNA by the ELPs protected siRNA from RNase A (Fig. 2b). Tat- A_4_V_48_ provided better siRNA protection than the other ELPs. The high number of positively charge arginines (R) present strengthened binding with siRNA and increased encapsulating capacity and siRNA shielding. E_28_ was not found to condense with nucleic acid, indicating that ELP does interact with nucleic acid but rather provides thermal sensitivity and stability.

For successful gene delivery, ELPs should undergo phase transition at a clinically relevant temperature and release gene cargoes at therapeutic site. The optical density measurements at different temperatures clearly indicated that condensation of siRNA with ELPs further reduced their transition temperatures (Fig. 2c–f). It was anticipated that positive charged amino acids residue presents in the polypeptides were neutralized by negative charged siRNA residues. Thus, ELPs remained in a highly protonated state and slightly depressed Tt values [45]. DLS particle size measurements confirmed the increased in particle sizes after nucleic acids encapsulation indicated the stable self-assembly of ELP variants into nanocomplexes, and this was further confirmed by TEM images. Complexations of siRNA with Tat-A_1_E_28_ or Tat-A_4_V_48_ resulted in increased surface charge versus Tat-A_1_E_28_ or Tat-A_4_V_48_ alone (Additional file 11: Table S1). Surface charge density studies have shown zeta potentials are positively related to the stabilities of many colloidal systems, and that more positive zeta potentials increase interactions with cell membranes [46]. Thus, the reduced zeta potential of siRNA/Tat-E_28_ suggests low transfection efficiency.

In order to determine the mechanism of gene delivery by ELPs, intracellular tracking of ELPs complexes consisting of labeled siRNA was visualized by confocal microscopy. Yellow spots resulting from overlapping green siRNA signals with red lysosome or endosome signals were more frequently observed for siRNA/Tat-A_1_E_28_ and siRNA/Tat-A_4_V_48_ than other complexes (Fig. 3b and Additional file 7: Figure S7). It appeared these complexes were internalized by tumor cells via IL-4R mediated endocytosis and then fused with endosomes followed by lysosome where complexes would be degraded and siRNA would be released to cytosol. Tat-E_28_ was taken up by cells at a lower rate and associated and co-localized less well with endosomes and lysosomes. This slow internalization of Tat-E28 indicated it was taken up by a mechanism other than receptor-mediated endocytosis. It has been reported that pretreatment of HeLa cells with sodium azide or deoxyglucose (to deplete ATP) and subsequent incubation with CPP functionalized ELP or co-incubation with sucrose and CPP functionalized ELP (e.g., Tat-ELP, MTS-ELP, or Antp-ELP) significantly inhibited polypeptide internalization, which indicated the importance of clathrin-mediated endocytosis [34]. Thus, decoration of ELP with IL-4R specific ligands and a cell penetrating peptide increased its cell-binding ability as compared with singly functionalized counterparts.

Furthermore, our in vitro gene silencing assay confirmed that AP1 containing targeting ELPs more efficiently delivered siRNA and so more repressed targeted gene expression (Fig. 4a–d). On the other hand, Tat- E_28_ and E_28_ had minimal effects on gene expression, which demonstrated the presence of AP1 increased cellular uptake and resulted in more effective siRNA deliveries. Furthermore, western blot confirmed that Tat-A_1_E_28_ and Tat-A_4_V_48_ mediated siRNA delivery maximally suppressed or enhanced gene expression. Furthermore, cell viability assays after siRNA/ELP complexes transfection produced no evidence of cytotoxicity, suggesting suitability for systematic administration (Additional file 8: Figure S8c).

Additionally, In vivo distribution studies showed that Tat-A_1_E_28_ and Tat-A_4_V_48_ delivered Cy5.5 labeled siRNA specifically into tumor tissues in 4T1-allograft murine model, whereas free siRNA and Tat-E_28_ mediated delivery resulted in minimal accumulation in tumor tissues and strong non-specific accumulation (Additional file 9: Figure S9). Ex vivo fluorescence images of tumors and organs isolated at 6 h post-injection confirmed that the IL-4R targeting ELPs displayed higher siRNA accumulations in tumors than E_28_ or Tat-E_28_ (Fig. 5a and b). Furthermore, E_28_, Tat-E_28_, and Tat-A_1_E_28_ showed higher accumulations in liver and kidneys, presumably due to rapid degradation. However, siRNA/Tat-A_4_V_48_ showed relatively less accumulation in vital organs possibly due to its greater molecular weight and number of targeting AP1 peptides. All four ELPs showed negligible accumulations in other organs (e.g., spleen, lungs, and heart). Immunohistological examination of tumor tissues confirmed that Tat-A_1_E_28_ and Tat-A_4_V_48_ effectively delivered siRNA into tumor tissues, possibly by IL-4R mediated endocytosis and siRNA release (Fig. 5c). After confirming enhanced siRNA accumulation by Tat-A_1_E_28_ and Tat-A_4_V_48_ in tumor tissues, we investigated the in vivo silencing of luciferase in the 4T1-luc allograft model. IV administration of siRNA/Tat- Tat-A_1_E_28_ or siRNA/Tat-A_4_V_48_ dramatically inhibited luciferase expression, whereas an insignificant reduction was detected after administering siRNA/Tat-E_28_ or free siRNA (Fig. 6a). These results along with our immunohistology findings of excised tumor tissues further demonstrated targeting ELPs can deliver siRNA more specifically to tumors highly expressing IL-4R by protecting payloads, traversing membrane barriers, and releasing their contents to silence target genes.

## Conclusion

We describe a non-viral, gene-delivery system comprised of elastin-like polypeptide, Tat, and AP1 with considerable potential as a gene therapy for cancer. It was observed that when bound to siRNA, these the ELPs self-assembled to form nanocomplexes that were stable under physiological conditions, possessed a positively charged surface, had particle sizes suitable for cell internalization, and specifically targeted IL4 receptor expressing tumors. This system was found to deliver plasmids harboring the siRNA selectively and effectively to tumor cells, and silence the gene of interest. We suggest the described ELP based systems offer a promising strategy for gene therapy applications that specifically target tumor cells expressing IL4R at high levels.

## Supplementary information

**Additional file 1: Figure S1.** The corresponding amino acid sequence of E_28_, Tat-E_28_, Tat-A_1_E_28_ and Tat-A_4_V_48_. Blue: cell penetrating peptide Tat, Red: IL-4R binding ligand AP-1.

**Additional file 2: Figure S2.** Characterization of purified ELP variants. The purities and molecular weights of purified proteins were determined by SDS-PAGE. Expected molecular weights of ELPs are follows, E_28_ (12.18 KDa), Tat-E_28_ (13.19 KDa), Tat-A_1_E_28_ (15.00 KDa), and Tat-A_4_V_48_ (26.81 KDa) (from right).

**Additional file 3: Figure S3.** Cell binding of ELP variants. MDA MB231 cells were incubated with Alexa Fluor 488 labeled ELPs for 1 h at 4 °C. After staining nuclei and membranes with Hoechst 33342 or WGA-Alexa 594, cell binding activities were determined by confocal microscopy. Scale bar, 20 µm.

**Additional file 4: Figure S4.** Cellular uptakes of ELP variants. MDA MB231 cells were incubated with Alexa Fluor 488 labeled ELPs for 1 h at 37 °C. After staining nuclei and membranes with Hoechst 33342 or WGA-Alexa 594, cellular uptakes of ELPs were determined by confocal microscopy. Scale bar, 20 µm.

**Additional file 5: Figure S5.** Cell binding by ELP variants. 4T1 cells were incubated with Alexa Fluor 488 labeled ELPs for 1 h at 4 °C. Cell binding activities of ELPs were determined by confocal microscopy. Cell nuclei and membranes were stained with Hoechst or WGA-Alexa 594. Scale bar, 20 µm.

**Additional file 6: Figure S6.** Cellular uptake of ELP variants. 4T1 cells were incubated with Alexa Fluor 488 labeled ELP variants for 1 h at 37 °C. After staining nuclei and membranes with Hoechst 33342 or WGA-Alexa 594, cellular uptakes were assessed by confocal microscopy. Scale bar, 20 µm.

**Additional file 7: Figure S7.** Intracellular tracking of siRNA/ELPs complexes. MDA MB231 cells were incubated with siRNA/ELPs complexes for 1 h at 37 °C and then stained with lysotracker. The co-localizations of siRNA/ELPs complexes with lysosomes were assessed by confocal microscopy. Representative confocal images from five experiments. Blue: nuclei stained with Hoechst 33342; Green: siRNA/ELPs complexes; Red: lysotracker. Scale bar, 10 μm.

**Additional file 8: Figure S8.** Luciferase gene silencing. (a–b) 4T1 cells (3X103) were plated in 96-well plates and treated with different concentrations of siRNA (50, 100, 200 nM) encapsulated with ELPs at 1:20 molar ratio. Gene silencing was examined by measuring BL (bio-luminescence) using IVIS (n = 3). (c) 4T1 cells (3X103) were plated in 96-well plates and treated with siRNA 200 nM encapsulated with ELP variants at 1:20 molar ratio for 48 h. Cellular viabilities were assessed by measuring WST-8 absorbance at 450 nm (n = 5 samples). The graph represents percentage of cell viability when compared to control non treated cells. The results are representative of 3 independent experiments.

**Additional file 9: Figure S9.** Mice bearing a subcutaneous 4T1 tumor were injected intravenously with Cy5.5 labelled siRNA encapsulated in ELP variants (molar ratio 1:20 (siRNA:ELPs)) at a siRNA dose of 250 µg/kg. The in vivo fluorescence images shown were taken at different times after injection using the IVIS in vivo imaging system. The results shown are representative of 3 independent experiments.

**Additional file 10: Figure S10.** Immunohistological staining of tumor tissue sections obtained after therapy. Nuclei were stained with Hoechst 33342 (blue), and luciferase expression on cells was visualized by anti-Luc antibody staining (green). The confocal images shown are representative of three experiments (scale bar = 20 μm).

**Additional file 11: Table S1.** Chemical characteristics of ELP variants before and after the encapsulations of siRNA.
