# Supplementary material for: Development of elastin-like polypeptide for targeted specific gene delivery in vivo
Source: J Nanobiotechnology. 2020 Jan 17;18:15. doi: 10.1186/s12951-020-0574-z (PMC6969399; doi:10.1186/s12951-020-0574-z)
Supplement: Supplementary file 10 — Additional file 10: Figure S10. Immunohistological staining of tumor tissue sections obtained after therapy. Nuclei were stained with Hoechst 33342 (blue), and luciferase expression on cells was visualized by anti-Luc antibody staining (green). The confocal images shown are representative of three experiments (scale bar = 20 μm). [file 12951_2020_574_MOESM10_ESM.docx]

**Development of elastin-like polypeptide for targeted specific gene delivery in vivo**

Aena Yi ^1^, Dahye Sim^1^, Young-Jin Lee ^1^, Vijaya Sarangthem ^1,2,^*, Rang-Woon Park ^1,^*

^1^Department of Biochemistry and Cell Biology, Cell & Matrix Research Institute, Kyungpook National University, School of Medicine, Daegu 41944, Republic of Korea.

^2^Department of Pathology, All India Institute of Medical Sciences, New-Delhi 110029, India.

* Corresponding authors. Telephone No.: +82 53 420 4822; Fax: +82 53 422 1466

E-mail address: [nwpark@knu.ac.kr](mailto:nwpark@knu.ac.kr) (R.-W. Park), [devi1703@gmail.com](mailto:devi1703@gmail.com) (V. Sarangthem)

**Methods**

**Materials**

Restriction enzymes (Bam HI, HinD III, Sfi I, Nde I, PflM I, and Bgl I), T4 ligase, and Calf intestinal phosphatase (CIP) were obtained from New England Biolabs Inc., pRSET b+ vector (Invitrogen Carlsbad, CA, USA) and pET 25b+ vector (Novagen Inc. Milwaukee, WI, USA) were used for cloning and expression. Competent E. coli DH5α and BL21 were purchased from Invitrogen, Carlsbad, CA, USA. E. coli was cultured in Circle Grow Media (MP Biomedicals, LLC) containing ampicillin 0.1mg/ml (Affymetrix, CA, USA). DNA oligonucleotides were synthesized and all DNA sequences were confirmed by DNA sequencing (Macrogen Inc., Korea). DNA miniprep and gel extraction kits were acquired from Elpis Biotech Inc., Korea.

Negative control siRNA (NC-siRNA), the luciferase siRNA- (sense-GGACGAGGACGAGCACUUCUU, and anti-sense-UUCCUGCUCCUGCUCGUGAAG were obtained from Bioneer Inc (Daejeon, Korea). Fluorescein isothiocyanate (FITC) and Cy 5.5 labeled NC-siRNAs (the 5`-end of the sense strand conjugated with FITC dyes) were obtained from Bioneer for *in vitro* uptake assay and *in vivo* biodistribution studies.

**Design and construction of ELP variants**

pET 25 b+ expression vector was modified by inserting annealed oligonucleotides coding for Tat-GGG and Tat-GGG-AP1-GGG genes containing Nde I, Sfi I and HinD III restriction sites. After confirmation by DNA sequencing, the modified pET25b+ vector was digested with Sfl I and then enzymatically dephosphorylated using CIP for 1 h. The [(VPGVG)_5_(VPGFG)_2-_(VPGVG)_3_(VPGGG)_3_(VPGAG)]_2_ gene generated by recursive directional ligation (RDL) and ligated with linearized modified pET- Tat-GGG and pET-Tat-GGG-AP1-GGG to obtain Tat-E_28_ or Tat-A_1_E_28_. For the multivalent targeting of ELP, A_4_V_48_ [(RKRLDRN-VPGVG _12_)_4_] was generated using the recursive directional ligation method and ligated with pET-Tat-GGG to obtain Tat-A_4_V_48_. Positive colonies were confirmed by restriction digestion using Nde I and HinD III and products were confirmed by gene sequencing (Macogen Inc. Seoul, Korea).

**Oligonucleotides sequences used to modify pET vector**

**1. Tat-GGG**

Forward - TATGAGCGGCTACGGCCGTAAAAAACGTCGTCAACGTCGTCGTGGC GGCGGGCCGGGCTGGCCGTGCTAAA

Reverse - AGCTTTTAGCACGGCCAGCCCGGCCCGCCGCCACGACGACGTTGACG ACGTTTTTTACGGCCGTAGCCGCTCA

**2. Tat-GGG-AP1-GGG**

Forward-TATGAGCGGCTACGGCCGTAAAAAACGTCGTCAACGTCGTCGTGGC GGCGGCCGTAAGCGTCTTGATCGGAATGGCGGCGGGCCGGGCTGGCCGTGCTAAA

Reverse-AGCTTTTAGCACGGCCAGCCCGGCCCGCCGCCATTCCGATCAAGACG CTTACGGCCGCCGCCACGACGACGTTGACGACGTTTTTTACGGCCGTAGCC GCTCA

Protein purification

Competent E. coli, BL21 (DE3) (Invitrogen, Carlsbad, CA, USA) were transformed with pET 25b+ vector containing E_28_, Tat-E_28_, Tat-A_1_E_28_ or Tat-A_4_V_48_ gene. For the preparation of starter culture, 20 mL of Circle grow media (MP Biomedicals, CA, USA) containing 0.1 mg/ml ampicillin (AMRESCO LLC, OH, USA) was inoculated with transformed E.coli and incubated overnight at 37 °C in shaking incubator. Cultures were further inoculated into 750 mL of fresh Circle grow media containing ampicillin and grown to an optical density (OD) at 600 nm of between 0.8 to 1.0. Then, 1 mM IPTG (Carbosynth Limited, Berkshire, UK) was added to induce protein synthesis. After 4 h of protein induction, cells were harvested by centrifugation at 4000 rpm for 20 min at 4 °C. The cell’s pellets were resuspended in lysis buffer containing 5% protease inhibitor (Sigma-Aldrich Co. LLC, USA), and subjected to sonication for 10 min. Cell lysates were centrifuged for 20 min at 12000 rpm in 4 °C, and phase transition of was induced by adding 1-4 M NaCl to supernatants. ELP pellets were collected after centrifuging at 12000 rpm for 20 min at room temperature. In the same way 3 rounds of inverse temperature cycling (ITC) was repeated to completely remove the cell debris. ELP concentrations were measured using a Cary UV-visible spectrophotometer (Agilent Technologies, CA, USA). The molecular weights and purities of proteins were confirmed by SDS-PAGE.

Confocal microscopy

MDA MB231 or 4T1 cells (8x10^4^) were seeded on 4-chambered slides and incubated for 24 h. The cells were then treated with 0.325 µM of Alexa Fluor 488 labeled ELPs for 1 h at 4 or 37°C. After several washed with PBS, the cells were fixed with 4% paraformaldehyde and cell nuclei were stained with Hoechst 33342, and cell membranes were stained with Wheat Germ Agglutinin Alexa Fluor 594 (Molecular Probes, Inc., Eugene). Cell binding efficiency of respective polypeptides was analyzed using a Zeiss LSM-510 Meta confocal microscope.

**Intracellular tracking**

To track lysosomes, MDA-MB231 (2X10^5^) cells were seeded on 20mm × 20mm coverslips on 6-well plates in Dulbecco Modified Eagle Medium. Negative control siRNA (NC-siRNA) of luciferase gene labeled with fluorescein was used to determine intracellular localization. siRNA/ELP variant complexes, including 50 pmol NC-siRNA were added to each well for 1 h or 4 h at 37°C. Pre-warmed media containing 50 nM of suspended LysoTracker (Molecular Probes, Life technologies, Eugene, OR, USA) was then added to each well and incubated for 3 h at 37 °C. Cells were then fixed with 4% paraformaldehyde, washed several times with PBS, and nuclei were stained with Hoechst 33342. Cells were then observed under a Carl Zeiss LSM700 Confocal microscope.

**Cell viability**

4T1 cells (5x10^3^) were seeded in 96 well plates, incubated for 24 h, and treated with siRNA/ELP variant complexes containing 200 nM of siRNA for 6 h. Media were then replaced with complete medium containing 10 % FBS. Cells were then allowed to grow for 48 h. Cell viabilities after siRNA/ELP variant complexes treatment were measured using a Cell Counting Kit-8 (CCK-8) (Dojindo Laboratories, Kumamoto, Japan). CCK-8 solution (10 µL) was added to each well, incubated at 37°C for 1 h, and absorbance was measured at 450 nm using a microplate reader.


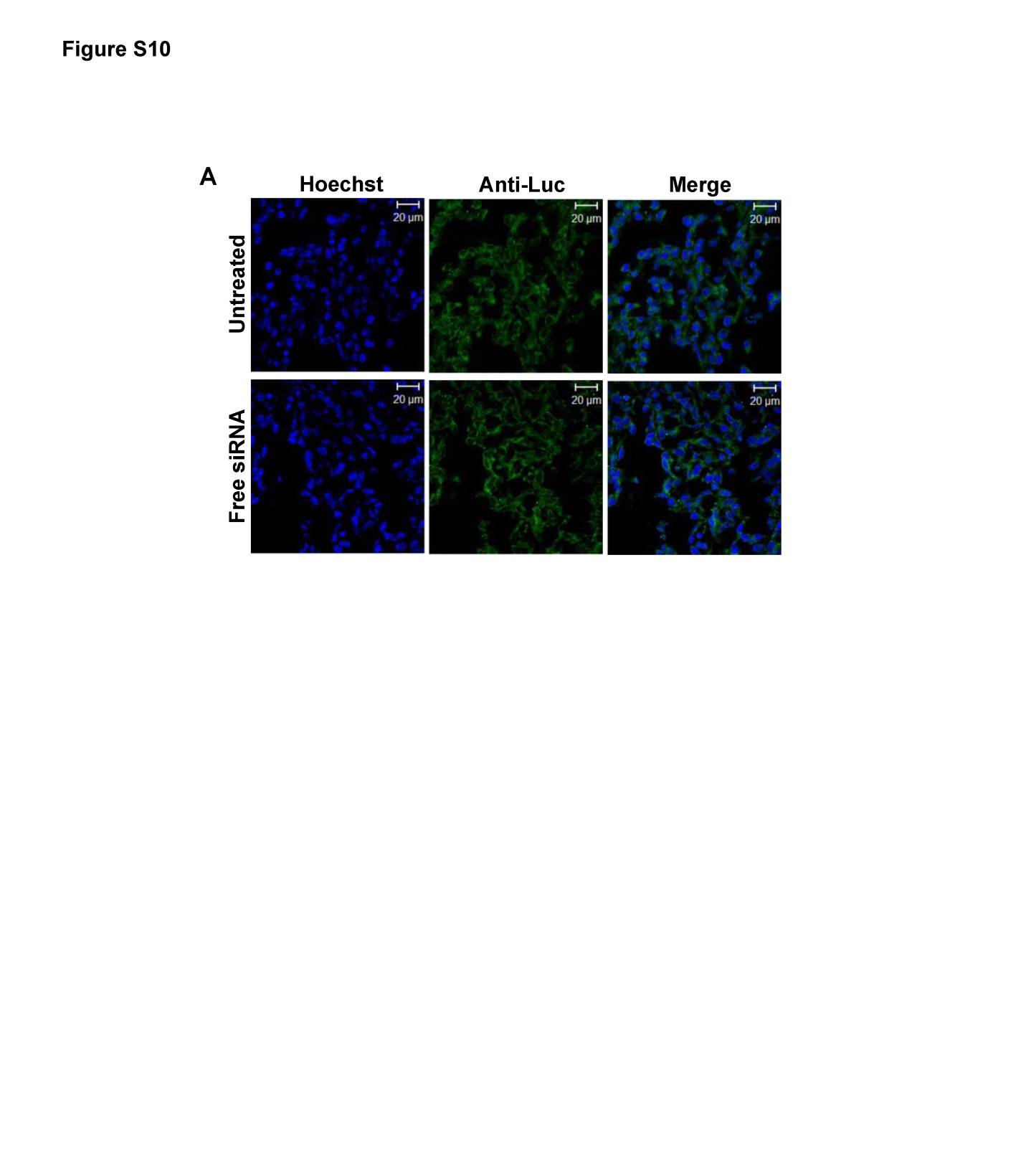


**Additional file 10: Figure S10.** Immunohistological staining of tumor tissue sections obtained after therapy. Nuclei were stained with Hoechst 33342 (blue), and luciferase expression on cells was visualized by anti-Luc antibody staining (green). The confocal images shown are representative of three experiments (scale bar = 20 μm).
